# Experience of quality management system in a clinical laboratory in Nigeria

**DOI:** 10.4102/ajlm.v1i1.18

**Published:** 2012-10-29

**Authors:** Rosemary A. Audu, Ugochukwu Sylvester-Ikondu, Chika K. Onwuamah, Olumuyiwa B. Salu, Fehintola A. Ige, Emily Meshack, Maureen Aniedobe, Olufemi S. Amoo, Azuka P. Okwuraiwe, Florence Okhiku, Chika L. Okoli, Emmanuel O. Fasela, Ebenezer. O. Odewale, Roseline O. Aleshinloye, Micheal Olatunji, Emmanuel O. Idigbe

**Affiliations:** 1 Human Virology Laboratory, Nigerian Institute of Medical Research, Lagos, Nigeria

## Abstract

**Issues:**

Quality-management systems (QMS) are uncommon in clinical laboratories in Nigeria, and until recently, none of the nation’s 5 349 clinical laboratories have been able to attain the certifications necessary to begin the process of attaining international accreditation. Nigeria’s Human Virology Laboratory (HVL), however, began implementation of a QMS in 2006, and in 2008 it was determined that the laboratory conformed to the requirements of ISO 9001:2000 (now 2008), making it the first diagnostic laboratory to be certified in Nigeria. The HVL has now applied for the World Health Organization (WHO) accreditation preparedness scheme. The experience of the QMS implementation process and the lessons learned therein are shared here.

**Description:**

In 2005, two personnel from the HVL spent time studying quality systems in a certified clinical laboratory in Dakar, Senegal. Following this peer-to-peer technical assistance, several training sessions were undertaken by HVL staff, a baseline assessment was conducted, and processes were established. The HVL has monitored its quality indicators and conducted internal and external audits; these analyses (from 2007 to 2009) are presented herein.

**Lessons learned:**

Although there was improvement in the pre-analytical and analytical indicators analysed and although data-entry errors decreased in the post-analytical process, the delay in returning laboratory test results increased significantly. There were several factors identified as causes for this delay and all of these have now been addressed except for an identified need for automation of some high-volume assays (currently being negotiated). Internal and external audits showed a trend of increasing non-conformities which could be the result of personnel simply becoming lax over time. Application for laboratory accreditation, however, could provide the renewed vigour needed to correct these non-conformities.

**Recommendation:**

This experience shows that sustainability of the QMS at present is a cause for concern. However, the tiered system of accreditation being developed by WHO–Afro may act as a driving force to preserve the spirit of continual improvement.

## Introduction

Laboratories play a pivotal role in all disease control and prevention programmes by providing timely and accurate information for patient management and disease surveillance.^1^ Clinical laboratories specifically, provide accurate diagnosis of present, recent or past infections for appropriate case management.^1^ Though laboratories form the backbone of health systems, providing health care workers with critical test results for numerous deadly diseases, yet in sub-Saharan Africa, which carries a huge disease burden, laboratories are among the world’s most ill-equipped and poorly resourced facilities. As of 2009, there were 340 diagnostic laboratories in sub-Saharan Africa that were accredited and 312 of these (92%) were found in South Africa.^2^ In Nigeria, there are 5349 diagnostic laboratories, of which only the Human Virology Laboratory (HVL) has ISO certification.

In 2000, the Ford Foundation provided a grant to the Nigerian Institute of Medical Research for the establishment of a National Reference Laboratory with capacities for basic, applied and operational research on HIV and AIDS in the country. The grant, which was worth $250 000, was meant for the construction of a laboratory facility, the purchase of equipment, and training of personnel. Subsequently, an additional grant of $150 000 was awarded for two years for sustainability activities, such as revolving funds for purchase of kits and reagents. Prior to this, there was no laboratory with facilities for HIV molecular diagnostics in Nigeria, requiring clinical samples from people living with HIV to be sent out of the country to laboratories abroad for relevant tests and analyses. With the grant funding, two new large laboratory facilities were built and fully equipped with state-of-the-art equipment for HIV and AIDS research and services.

Setting up the Quality Management System at the HVL involved two major stages, namely, planning and implementation. The planning stage involved identifying and mapping out processes, risk assessment of the processes and appropriate management, as well as identifying quality indicators and setting targets for monitoring. Implementing this process approach to management facilitated bottom-up communication, ownership and participation, thereby improving the quality of services provided.

In order to meet international standards, the laboratory worked to conform to the requirement of NIS ISO 9001:2000 and has in fact converted to the ISO 9001:2008 standard, making it the first diagnostic laboratory to be so certified in Nigeria. The major difference between the two standards is that the ISO 9001:2008 standard has a broader interpretation and application of its requirements. Because ISO 15890 requirements are more relevant to diagnostic laboratories, the HVL has applied for the WHO accreditation scheme through the efforts of the President’s Emergency Plan for AIDS Relief (PEPFAR), which uses this standard.

The effort of obtaining certification originated with the then-Director General, a fact which facilitated securing resources and approvals. Despite the high pre-assessment non-conformities, this ‘buy-in’ from staff at the highest levels of management was key to the success of this endeavour. Implementing the QMS was not without its challenges, however. One major challenge was getting the other staff to buy into this vision. This was achieved through involving every staff member, regular awareness creation at staff meetings, ensuring good staff morale and recognition of staff for exemplary performance.

There is a clearly defined need for attracting and retaining qualified, motivated personnel within the public health sector. In the HVL, the majority of laboratory personnel were either recent graduates completing compulsory service to the nation or on internships with the laboratory and, as such, they typically maintained a high level of performance. A few of the longer-serving staff were determined to be performing at an unsatisfactory level and were posted out of the unit. This reduced the staff to 7 research fellows, 12 laboratory scientists and 12 support staff of varying cadres.

An important aspect of addressing shortcomings in staff performance was the requirement that all personnel undergo basic training in performance standards. Initially, documentation of non-conformities among staff was challenging as this was frequently misunderstood as a means of ‘reporting an individual’ for an infraction. Staff members were informed that this documentation was not for any punitive purpose but was intended as a means of tracking and improving the system. The need to address this misperception among staff has been ongoing.

Another factor related to laboratory staffing had to be addressed as well. Though Nigeria, unlike many African nations, is not facing a crisis with respect to shortage of health workers, ‘brain circulation syndrome’ is quite high, with personnel frequently moving from one job to another in search of better wages or conditions of service. The HVL employed a group of highly skilled and dedicated staff who were committed to the vision of adopting the QMS and working toward accreditation after having undergone several training sessions on QMS. However, because of the HVL’s unique status as a self-sustaining laboratory within the Government establishment, personnel were not guaranteed job security and this fact adversely affected the sustainability of the laboratory. Moreover, the WHO recommends training of health workers to ensure quality service delivery and the retention of these trained workers because essential health services cannot be provided by people working on a voluntary basis if the service is to be sustainable. It is also recommended that trained health workers who are providing essential health services receive adequate wages and appropriate and commensurate incentives.^3^ Hence, the need to ‘absorb’ HVL’s staff as permanent Ministry of Health employees was identified as a solution to this issue. In July 2008, 20 project staff of the HVL were absorbed as permanent Government staff and the laboratory was made a unit in the Microbiology Division of the Nigerian Institute of Medical Research (NIMR), which is a parastatal institution under the Ministry of Health.

An additional challenge was the lack of a basic quality manual to establish guidelines for the HVL that were codified and easily accessible for personnel. With assistance from a trainer from the Standard Organisation of Nigeria (SON), this quality manual was eventually developed and adopted by the laboratory. The quality manual, which was written in line with the requirements of the standard, contained the structure of the quality system and its policies. But because SON was not typically involved with developing capacity for QMS in clinical settings, this made the overall expenditure on training higher, because assistance had to be sought first from Senegal.

Similarly, the laboratory found it necessary to adopt a quality policy which drives its vision as well as to establish a Top Management Committee, in line with the ISO requirement, which serves as the Management Advisory Committee to the laboratory.

## Description

In November 2005, two personnel from the HVL were sent to the BIO 24 laboratory in Dakar, Senegal, which was an ISO 9001:2000 certified clinical laboratory, to study the implementation of a QMS there. By July 2006, a consultant from Senegal and the laboratory manager from the certified laboratory had trained the HVL staff on a Basic Implementation Course, and a baseline assessment had been conducted. The laboratory then defined its processes and began to implement some basic QMS structures such as developing of policies, proper archiving of records, and selection of quality indicators for monitoring the system. We also introduced regular and quality-focused staff meetings, which included training and retraining.

By June 2007, the SON was identified as an appropriate organisation to train staff on the ISO 9000 Basic and Auditors’ Course; 13 staff members qualified as internal auditors as a result of this training. Twenty staff members were again trained on ISO/IEC 17025 in August 2008 and later undertook the ISO 15189:2007 Laboratory Accreditation and Auditors’ Course in March 2009. Both of these training courses were also conducted by the SON. The laboratory also successfully completed a revalidation course for ISO 9001:2008.

Though the process of obtaining certification and ultimately accreditation is an expensive one, the HVL project was able to address some of the costs through income generated from the services rendered by the laboratory. Because NIMR management saw the value of the improvements in laboratory personnel performance, the resources required have been perceived as a worthy investment and provided to continue the process.

Since 2007, the HVL’s quality indicators have been monitored, customer surveys have been performed, and internal and external audits have been conducted as part of the ongoing process. Various methods were used for the customer survey conducted twice a year. The commonest method was the use of questionnaires administered to patients, clinicians and other laboratories that patronised the HVL. These questionnaires were designed and analysed by staff members. Other occasional methods used included in-depth interviews with clinicians, focused group discussion with patients and the use of questionnaires administered to suppliers. Suggestions from these surveys were used to improve the system. Some staff of the laboratory who had qualified as internal auditors carried out internal audits twice a year. They prepared checklists using the ISO 9001:2000 standards and the laboratory quality manual; corrective actions were taken for all reported non-conformities. External audits were however carried out twice a year by the Standards Organisation of Nigeria to ensure the QMS was still functional. This is a mandatory requirement, if the certification was to be maintained. Reports of all of these activities were presented to the management during annual reviews.

## Lessons learned

By December 2007, the laboratory was deemed to be in compliance with the ISO 9001:2000 standard and was awarded its certificate in February 2008. By 2010, the HVL had conformed to ISO 9001:2008. The quality indicators for the operational processes and the audit analyses from 2007 to 2009 are presented below.

Table 1 shows the performance of some indicators in the operational system for all assays within the period reported. There was an improvement in the indicators for the pre-analytical and analytical processes over the years. It was observed earlier that there was a gap in awareness on how to collect samples for the various tests that were rendered in the laboratory. Continuous education is needed for all stakeholders involved in laboratory testing to improve the quality of the pre-analytical phase of the total-testing process.^4^ The laboratory prepared a clients’ handbook which contained information on time of operation, test offered, specimen type, test turnaround time, packaging, storage and transportation of samples. This was used to educate clinicians and other laboratory scientists who send samples to HVL. These clients were also invited to the laboratory and were further trained on proper sample collection, storage and transportation. There was a continuous decline in the number of unanalysed samples and external controls exceeding specification, which is a positive development for the QMS. Westgard rules applied to a Levey-Jennings chart as practiced in the HVL are programmed to determine when an analytical run should be rejected. These rules are applied carefully so that true errors are detected while false rejections are minimised. The rules applied to high volume chemistry and hematology instruments produce low false rejection rates.^5^ This was achieved in the HVL; however, values recorded for the external controls were higher than values reported in other studies,^6^ hence more rigorous monitoring is required. During the monitoring, it was observed that a few personnel still had challenges in using the tool while another staff member had not fully understood the benefit of complying strictly with the rules, hence training and retraining on application and benefits of the Westgard rule is now a regular practice in the HVL. There was an improvement in maintaining environmental temperatures within acceptable ranges. Scores obtained from participation in external quality assessment were within an acceptable range.

**TABLE 1:** Performance of some indicators in the operational system between 2007 and 2009 for all assays in HVL.

Operational system	Performance indicators	Years					
		2007		2008		2009	
		*n*	(%)	*n*	(%)	*n*	(%)
**Pre-analytical**	Sample rejection	35	1.6	15	0.1	28	0.1
	^‡^Sample with wrong specification	88	15.7	54	0.2	19	0.1
	Loss of samples	71	0.3	12	0.1	14	0.1
**Analytical**	Unanalysed samples	^†^NA	-	76	0.3	2	0.01
	External control exceeding specification	4 868 (21.6)	-	1 756 (7.2)	-	1 465 (6.2)	-
	Maintaining environmental temperatures within acceptable ranges	-	57.4	-	84.4	-	85.8
	Average performance in external quality assessment	-	98.3	-	96	-	95
**Post-analytical**	Delay of results	300	1.3	369	1.5	4 294	18.3
	Error in data entry	158	0.7	13	0.1	15	0.1
**Total number of samples received in the year (*N*)**		**22 559**	-	**24 392**	-	**23 478**	-

† Records are not available for unanalysed samples for the year 2007.

‡ This indicates when the sample was not appropriate for the test requested.

An improvement was also observed in the reduction of errors in data entry in the post-analytical process (Table 1). The delay in reporting results became significantly higher in 2009 in spite of the fact that implementing the QMS is expected to improve turnaround time.^7^ Determining the root cause of this delay in order to eliminate it is a necessary approach.^8^ Several factors contributed to the delay of results such as issues in supply chain management (unavailability of test kits provided by partners), failure of quality control for two assays (Westgard rules), network problems (support infrastructure), and staff rotation. Another major challenge was the daily manual testing of large numbers of samples for some of the assays. Because the unavailability of kits was beyond the control of the laboratory, discussions were held with the supporting partner providing the kits to correct the challenges faced with supplies. There was also a change required in the brand of test kit because of repeated failure of the internal quality control, and an agreement for the maintenance of the database network was signed. In terms of staff rotation, an improved competency evaluation was established. Appeals are still being made to the supporting partner for automation of these assays particularly in a reference laboratory such as the HVL.

Comparison of internal audits by staff and external audits by SON over the three years showed an increase in the number of non-conformities, from 157 to 192 for internal audit and 39 to 55 for external audits – perhaps an indication that laboratory personnel were becoming lax in implementing the system. It is presumed that the process of applying for laboratory accreditation with its stringent technical demands could stimulate staff to sustain and even improve the system.^9^ This is most likely because of the stepwise accreditation preparedness approach of the World Health Organisation Regional Office for Africa (WHO-Afro). This programme is intended to provide an interim pathway for measuring, monitoring and recognising improvement toward the realization of international laboratory standards and subsequent application to full ISO 15189 accreditation.^2^

## Recommendation

For QMS to be implemented effectively there must be support from the highest levels of management; therefore strong advocacy to management is a necessary starting point. For laboratories planning to obtain accreditation, considerable training is required and this must be taken into account. Automation of laboratory systems has advantages such as reduction in staff time and supply costs, reduced turnaround times, as well as reduction in operational errors .^6^ Therefore it is recommended that clinical laboratories performing high volume testing in Africa adopt automation of their systems as they prepare for possible WHO assisted approach for laboratory accreditation.

The HVL audit experience shows there was an improvement in 2008 – the year that certification was obtained – but that, soon after, these gains diminished, suggesting that sustainability will continue to be a major challenge. The WHO’s accreditation preparedness scheme for African medical laboratories utilises a tiered system under which laboratories complying with the ISO 15189 standards are audited and awarded recognition from one to five stars (in the form of a certificate). This may serve to provide the necessary impetus for continual quality improvement for African laboratories. Under this program, audits are conducted annually and laboratories that fall behind will have their certificate of recognition withdrawn. Only a few of Africa’s laboratories are currently accredited to international standards, but, through this five-star accreditation preparedness process, laboratories will be able to be recognised for their improvement efforts using the WHO AFRO SLIPTA Checklist – and eventually apply for accreditation by a nationally-, regionally-, or internationally-recognised accreditation body. This is critical because good laboratory support leads to better health care delivery through correct diagnoses of diseases and monitoring of drug resistance. This is crucially important in Nigeria (and across Africa) to monitor patients infected with HIV, TB, malaria, and a host of other diseases. In addition, an efficient laboratory also can dramatically reduce waiting time to get results – allowing patients who often travel a day or more for testing to receive the laboratory results sooner. Studies have shown that when patients need to return for a second visit to a hospital or clinic for test results, significant percentages fail to do so.^10^

In spite of the challenges inherent in this process and the continued need for improvement, the HVL can attest that implementing QMS has brought about reliability and reproducibility of results as confirmed by its numerous clients. Also there has been a widespread recognition of the laboratory such that corporate and private clients seek services, which has resulted in improved profit. Consequently, most of the assays have now been automated. The introduction of the QMS in Nigerian medical laboratories – and in Africa generally – truly represents a major step forward and the beginning of a whole new era for the continent’s public health systems.
